# A novel methodology utilizing microchip implants to monitor individual activity and body temperature for assessing knee pain in group-housed rats

**DOI:** 10.1038/s41598-024-67024-7

**Published:** 2024-07-23

**Authors:** Shoichi Hasegawa, Riko Yamashita, Yusuke Nakagawa, Kazumasa Miyatake, Hiroki Katagiri, Tomomasa Nakamura, Hideyuki Koga, Ichiro Sekiya, Toshitaka Yoshii, Vicki Rosen, Kunikazu Tsuji

**Affiliations:** 1 Department of Joint Surgery and Sports Medicine, Tokyo Medical and Dental University, Tokyo, Japan; 2 Center for Stem Cell and Regenerative Medicine, Tokyo Medical and Dental University, Tokyo, Japan; 3 Department of Orthopaedic Surgery, Tokyo Medical and Dental University, Yushima 1-5-45, Bunkyo-ku, Tokyo, 113-8519 Japan; 4 Department of Developmental Biology, Harvard School of Dental Medicine, Boston, MA USA

**Keywords:** Biological techniques, Behavioural methods

## Abstract

The pain assessment in animals is challenging as they cannot verbally express the site and severity of pain. In this study, we tried a small implantable actimeter, “Nanotag”, to monitor spontaneous locomotor activity and body temperature in animals suffering from a chemical-induced rat knee arthritis as compared to naïve and steroid-treated rats. Nanotag could detect the decrease in locomotor activity quickly after the arthritis induction and anti-inflammation analgesic treatment by intra-articular injection of steroid significantly improved locomotor activity. These changes were in the same line with those of a conventional knee pain evaluation method (incapacitance test). Nanotag can be utilized as the non-interventional, continuous, and completely objective monitoring the amount of pain in rat knee arthritis model. This traditional yet innovative method may be universally applicable to various pain models and species, making it a worthwhile device for research across diverse fields.

## Introduction

As persistent pain is the greatest complaint in patients with musculoskeletal disorders, development of effective treatments for alleviating pain is an urgent issue. Experimental animal models are essential for the analysis of molecular mechanisms of pain development and its persistence, and various pain assessment techniques have been developed and studied in these models^1,2^. However, quantitative evaluation of pain in animals is still quite challenging, and several problems to accurate pain assessment remain. Firstly, animals do not verbally express the severity and the site of pain in a clear way. Secondly, it is quite difficult to determine the extent to which central nervous system effects, i.e., emotional influences such as social isolation or repeated physical restraints during measurement, are involved in causing pain due to peripheral nerve sensitization. Thirdly, pain assessment is usually performed during daylight hours when rodents are normally in a sleeping period. In addition to these technical difficulties and experimental procedural non-optimality, there is a concern that the subjective feelings of the experimenters may also influence the measurement results.

To overcome these difficulties and perform quantitative, reliable, and reproducible evaluation of pain, we tried a small implantable actimeter, “Nanotag”, to monitor spontaneous locomotor activity and body temperature in animals suffering from a chemical-induced rat knee arthritis as compared to naïve and steroid-treated rats. In recent years, various home cage analysis systems have been reported to measure the amount of locomotor activity in animals without distress^3–6^. However, there are only a limited number of tools that can relatively inexpensively measure the amount of locomotor activity and body temperature of many animals at once. Furthermore, there have been few studies using this method to measure pain avoidance behavior.

In this study, we showed that the device, Nanotag, could clearly detect the decrease in locomotor activity quickly after the arthritis induction and anti-inflammation treatment by intra-articular injection of steroid significantly improved it. These changes were in the same line with those of a conventional knee pain evaluation method such as incapacitance test (weight bearing imbalance). These data indicated that Nanotag can be utilized as the non-interventional, continuous, and completely objective monitoring the amount of pain in rat knee arthritis model. This traditional yet innovative method may be universally applicable to various pain models and species, making it a worthwhile device for research across diverse fields.

## Methods

### Ethics

This study was reported in accordance with ARRIVE guidelines. This study was approved by the Institutional Animal Care and Use Committee (IACUC) of Tokyo Medical and Dental University (Approval Number: A2022-104A).

### Animals

Male Wistar rats (Charles River, Tokyo, Japan) aged 9–10 weeks and weighing 310–350 g were purchased from Oriental Yeast Co., Ltd. (Tokyo, Japan). Two rats per cage were maintained in an animal facility with a temperature of 24–25 °C and humidity of 50–60% under a 12/12 h light/dark cycle with free access to food and water. The rat cages were cleaned once a week. Except for the provision of water, changing bedding, and conducting the experiments (intra-articular injections), the rats were not affected. Before the experiments, the rats were given one week to acclimatized to their environment.

### Materials

Monoiodo-acetic acid (MIA) and paraformaldehyde (PFA) were purchased from Sigma-Aldrich (St. Louis, MO, USA). Betamethasone (BM) was purchased from Shionogi Pharma Co., Ltd. (Osaka, Japan). Phosphate buffered saline (PBS) was purchased from Thermo Fisher Scientific (Waltham, MA, USA). Ethylenediaminetetraacetic acid (EDTA), Fast Green FCF, and isoflurane were purchased from Wako Pure Chemical Industries Ltd. (Osaka, Japan). Safranin-O was purchased from Chroma-Gesellschaft-Schmidt & Co. (Stuttgart, Germany). The anti-calcitonin gene-related peptide (CGRP) antibody was purchased from Peninsula Laboratories, LLC (San Carlos, CA, USA). Mayer's hematoxylin and 1% eosin alcohol solutions were purchased from Muto Pure Chemicals Co., Ltd. (Tokyo, Japan).

### Nanotag

**Figure 1:**
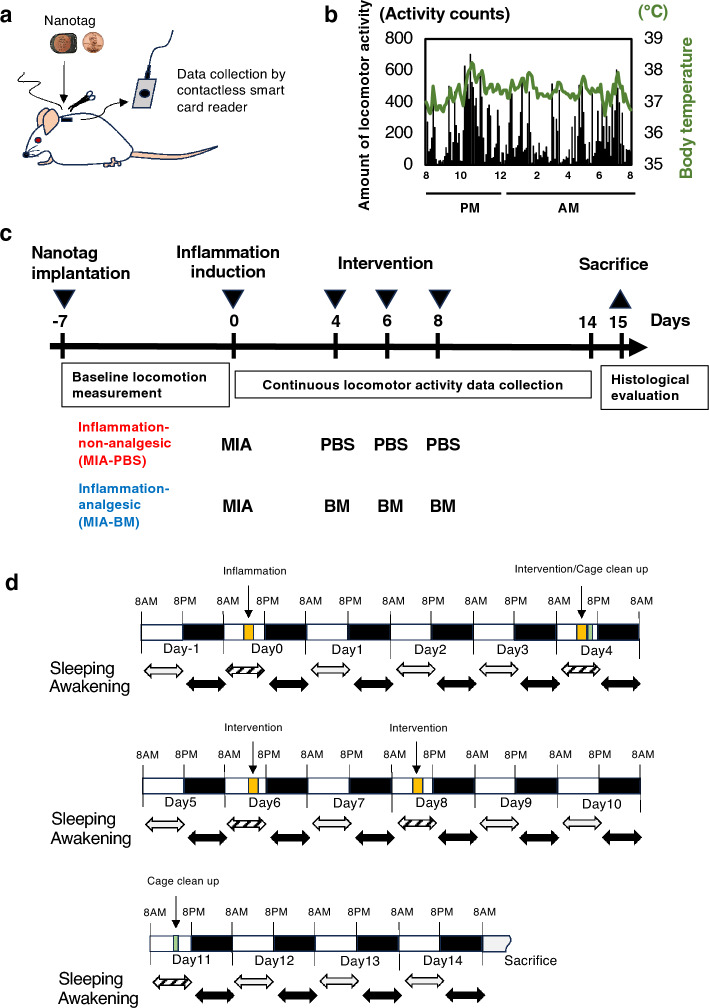
Experimental design and procedure. (**a**) Nanotag was implanted under the skin on the dorsal side of the rat neck. Activity counts and body temperature were automatically recorded for approximately 60 days and can be retrieved percutaneously into the computer by a non-contact reader. (**b**) Representative results of the measurements. (**c**) Schematic diagrams of experimental design. (**d**) Schematic diagrams of data collection. Data during intervention operations, such as injection and cage cleaning were excluded from the analysis (Hatched double arrows).

Nanotag is a small body-implantable actimeter (Kissei Comtec Co., Ltd. Tokyo, Japan, Fig. 1a). The size of this device is 18.8 mm in length, 14.2 mm in width, 7.1 mm in height, and 2.7 g in weight. This device has a 3-axis accelerometer (capacitive accelerometer) and a temperature sensor, and those sensors continually detect animal’s activity and body temperature without the need for restraint by the experimental subject (Fig. 1b). The locomotor activities of the animals were determined by the XYZ composite wave of the 3-axis accelerometer crossing the threshold from bottom to top per unit time was counted as the amount of locomotor activity, and the amount of locomotor activity recorded could be customized for measurement at any interval from 12 s to 5 min. In this study, we used the recommended threshold values proposed by the manufacturer, and the recording interval was set to every 5 min. With these measurement settings, the device can be used for up to 60 days. All detected data was stored into internal memory for retrieving wirelessly later (http://www.sleepsign.com/nanotag/index.html). Nanotag Viewer software provided by the manufacturer was used to control the device including measurement mode setting, initialization, measurement start, stop, data reading, and data processing. A contactless smart card reader, FeliCa (ISO/IEC 18,092), was used for data reading. By subcutaneously embedding the Nanotag with the antenna facing outward, data could be retrieved. In this study, data collection during the experimental phase was abstained from to minimize unnecessary interaction with the animals. Instead, all data spanning the entire duration were acquired post-experiment, subsequent to the euthanization of the animals. The use of Nanotags allowed for simultaneous measurement of multiple animals within the same cage.

### Implantation of Nanotag on the dorsal side of the rat neck

After 1 week of environmental adaptation, Nanotag insertion surgery was performed on the rats. Nanotag was set to automatically record locomotor activity and body temperature at five-minute intervals before surgery. The rats were anesthetized using 2% isoflurane in 2 L/min oxygen flow. Nanotags were sterilized by immersion in 2% povidone-iodine for 5 min immediately before implantation. The skin on the dorsal side of the neck was incised laterally, and the subcutaneous tissue was peeled away to create space. After subcutaneous implantation, the device was stitched and secured to the extramuscular membrane using a 5–0 nylon thread. Then skin was sutured. No complications or signs of severe infections were observed.

### Measurement of locomotor activity and body temperature using Nanotag

As a potential pain assessment method, we evaluated a small implantable actimeter, Nanotag, using a rat MIA–induced arthritis model^7–10^. MIA is a haloacetic acid in which one iodide atom takes the place of a hydrogen atom in the methyl group of acetic acid. Previous studies indicated that intra-articular injection of MIA induces severe joint inflammation followed by articular cartilage degradation in rodents. Thus MIA-induced arthritis model has been used in many studies for the analyses of joint pain and as a non-surgical osteoarthritis model. In this study, we employed BM as a sedative to assess the potential of Nanotag in gauging the efficacy of pharmaceutical intervention for knee pain. BM is a synthetic corticosteroid with anti-inflammatory and analgesic properties and is used as an intra-articular injection formulation for the treatment of arthritis^11,12^.

Nanotags were implanted subcutaneously in the dorsal side of rat necks 1 week before intra-articular injection of MIA or PBS (Fig. 1a,c and supplementary Fig. 1a)^13^. After Nanotag implantation, rats were allowed to recover for 1 week before being divided into three groups: the non-inflammatory control group (PBS-PBS, n = 18), the inflammatory–non-analgesic group (MIA-PBS, n = 18), and the inflammatory-analgesic group (MIA-BM, n = 18) (Fig. 1 and supplementary Fig. 1). Our previous study indicated that infrapatellar fat pad (IFP) fibrosis, which causes persistent pain, occurs around 5–7 days after knee joint inflammation. Thus, BM (MIA-BM group) or PBS (MIA-PBS group) were injected into the knee joints on days 4, 6, and 8^7–10^. On day 0, MIA (1 mg in 20 μL of PBS) was administered to both knee joints to induce joint inflammation. On days 4, 6, and 8, the PBS-PBS and MIA-PBS groups received an intra-articular injection of PBS (20 μL), whereas the MIA-BM group was injected with BM in both knee joints (80ug in 20 μL of PBS). Except on the days of intra-articular injection and cage cleaning, the rats were kept in a state of complete non-interference. Two rats were housed in each cage to eliminate the effect of the isolation-induced loss of locomotor activity^13^. Continuous measurements of locomotor activity were performed during the experimental period and data were stored in the internal memory of Nanotag. All data spanning the entire duration were acquired post-experiment and analyzed separately in the sleeping and awakening period (Fig. 1d).

### Data analysis (Locomotor activity counts, intensity, and body temperature)

Data recorded in the Nanotag were transferred using a contactless smart-card reader. Locomotor activity counts and body temperature were recorded every 5 min in CSV format (representative results are shown in Fig. 1b). Of note, data from three of 18 rats in MIA-BM group were not able to be used for further analysis because of the failure in data retrieval with unknown reason.

- Evaluation of total locomotor activity counts during the awakening and the sleeping period
  To evaluate the relative activity levels between the experimental groups, the total activity counts were calculated every 5 min for each rat (Supplementary Fig. 2a). Data were collected according to the schedule described in Fig. 1c. Data were analyzed separately for the awakening period (8 PM–8 AM) and the sleeping period (8 AM–8 PM) as described in Fig. 1d.
  1. Calculation of baseline value (pre inj).
    Because large individual differences in basal activity counts between rats were observed, daily activities were normalized using the average daily activity counts for the week from Nanotag implantation (day-7) to the day before joint inflammation induction (day-1) as indicated in Fig. 1c and Supplementary Fig. 2a.
  2. Calculation of relative activity level (Supplementary Fig. 2a).
    Relative activity level of each rat was calculated using the equation indicated below.
    [Relative activity level (fold) = total activity count of the day/average total activity count during pre-injection].
    The relative activity levels were calculated separately for each rat. Data are presented as mean and SD values of all rats. Numerical data are presented in Supplementary Table 1. Data collected during intervention operations, such as injection and cage cleaning, were excluded from the analysis (double arrows in Fig. 1d).
- Classification of activity intensity and assessment of changes over time in each activity intensity (difference in the number of epochs from day 0 [Awakening period; day 0 vs each day] or from day 1 [Sleeping period; day 1 vs each day])
  Because the Nanotag device can record patterns of locomotor activity, we defined locomotor activity thresholds of 300 and 150 every 5 min, as per prior studies, and classified them as follows: immobile (0), mild (1–150), moderate (151–300), and highly active (> 300)^13^. The number of epochs of locomotor activity was counted for each activity intensity. The intensity of activity every 5 min was categorized, and the total epoch numbers of each category for 12 h were counted. The difference in the number of epochs from day 0 was calculated as depicted in Supplementary Fig. 2b.
- Evaluation of time course changes in body temperature
  The body temperature of each rat was automatically recorded every 5 min using Nanotags. The average body temperature was calculated as the mean and SD (Supplementary Fig. 2c).

### Incapacitance test

To assess the weight-bearing imbalance between the left and right legs, only the right knee was subjected to inflammation induction and intervention. The left knee was kept intact throughout the experiment. The rats were divided into three groups: PBS-PBS, MIA-PBS, and MIA-BM groups (n = 12 in each group). Experimental procedures, such as MIA and BM/PBS injections, were the same as those indicated in Fig. 1c and supplementary Fig. 1a. Using an incapacitance tester (Linton Instrumentation, Norfolk), weight-bearing imbalance was assessed at the following time points: day 0 (pre-injection) and days 1, 4, 8, 10, and 14 after MIA injection^7,9,10^. The rats were acclimated to the tester for 3 days before MIA injection. The rats were placed in a clear plexiglass case for 5 min until the left and right limbs settled on independent force plates. All measurements were performed 100 times. The percentage of the weight-bearing imbalance in the ipsilateral hind limb was determined using the following equation, as previously described:^14^.

$$\begin{aligned} [{\text{weight distribution of}}\,{\text{the ipsilateral hind limb rate }}\left( \% \right) = & {\text{ipsilateral weight}}/\left( {\text{ipsilateral weight}} \right. \\ & \left. { + \,{\text{contralateral weight}}} \right) \times {1}00]. \\ \end{aligned}$$

### Immunohistochemical evaluations

Midsagittal sections were deparaffinized using xylene, rehydrated with graded alcohol, and soaked in PBS before immunohistochemical staining for CGRP-positive nerve fibers. Endogenous peroxidase activity was quenched using methanol and 0.3% hydrogen peroxide for 15 min. To enhance antigen retrieval, the sections were fixed in 4% PFA for 15 min, treated with Target Retrieval Solution (Dako, Glostrup, Denmark) in citrate buffer at 98 °C for 30 min, and then incubated for 20 min at room temperature. To avoid non-specific antibody binding, the sections were blocked with 5% normal goat serum for 30 min at room temperature (Vector Laboratories, Burlingame, CA, USA). A rabbit anti-CGRP polyclonal antibody was used as the primary antibody and incubated overnight at 4 °C (1:250 dilution in PBS containing 1% bovine serum albumin [BSA]; Peninsula Laboratories LLC, San Carlos, CA, USA). Sections were then incubated with biotinylated goat anti-rabbit Immunoglobulin G secondary antibody and visualized using Vectastain ABC reagent (Vector Laboratories). Hematoxylin was used for counterstaining, and the number of CGRP-positive fibers was counted according to previously established methods (n = 3, Fig. 3a)^7,9,10^. The evaluations were performed by three independent researchers in a blinded manner. Inter-experimenter co-efficiency (ICC2.1) was 0.95 (95% CI 0.82–0.99)^15,16^.

### Histological evaluations

Dissected knee joints were fixed in 4% paraformaldehyde at pH 7.4 for one week, then demineralized in 20% EDTA in PBS (pH 7.4) for three weeks, and embedded in paraffin. Sagittal sections were prepared with a thickness of 5 µm, de-paraffinized, and stained with hematoxylin/eosin (H/E) or Safranin-O/fast green.

To semi-quantitatively assess the severity of synovial inflammation, IFP inflammation grading was determined using the mid-sagittal section where the anterior cruciate ligament was visible^17^(n = 6, Fig. 3b). The evaluations were performed by three independent researchers in a blinded manner. Inter-experimenter co-efficiency (ICC2.1) was 0.73 (95% CI 0.45–0.90)^15,16^.

Cartilage degeneration was evaluated according to the OA Research Society International (OARSI) Grading System as previously reported^17,18^. Two sagittal sections of medial condyle at intervals of 200 µm were subjected for evaluation (n = 6, Fig. 3c). The evaluations were performed by three independent researchers in a blinded manner. Inter-experimenter co-efficiency (ICC2.1) was 0.95 (95% CI 0.87–0.98)^15,16^.

### Statistical analysis

For statistical evaluations, easy R (EZR) interface was used^19^. The evaluations of body temperature and histological analysis were performed using a *T*-test. To evaluate alterations in locomotor activity counts and intensity over time between the two groups, the average area under the curve (AUC) for each group (MIA-PBS and MIA-BM) was calculated to assess the cumulative effect across the observation period. First, the AUC was delineated for each day and group, followed by the calculation of the cumulative area up to each day. Subsequently, these cumulative areas were compared using a *T*-test. Differences were considered statistically significant at *P* < 0.05.

To assess inter-observer errors, intra-class correlation coefficients were used^15,16^. The ICC values were categorized as follows: poor reliability for values ≤ 0.5, moderate reliability for values between 0.5 and 0.75, good reliability for values between 0.75 and 0.90, and excellent reliability for values ≥ 0.90.

## Results

### Monitoring of spontaneous locomotor activity and body temperature in animals suffering from a chemical-induced rat knee arthritis by a small implantable actimeter, Nanotag

**Figure 2:**
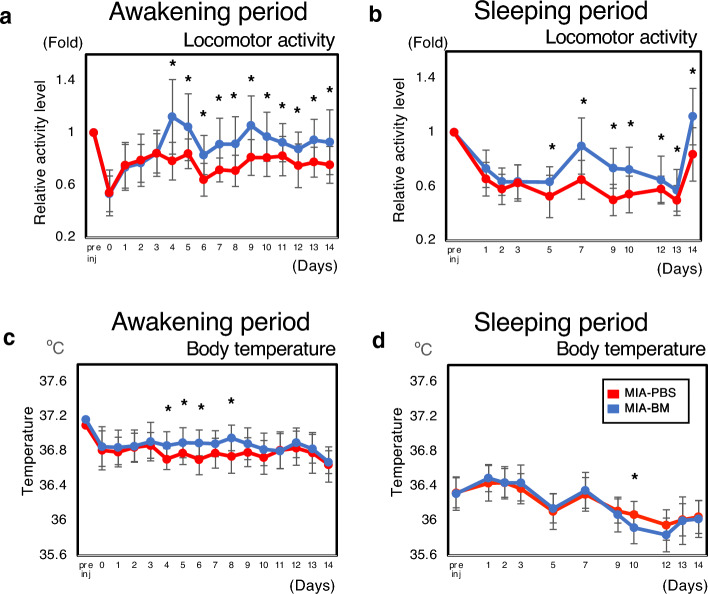
Measurement of locomotor activity and body temperature using Nanotag. (**a**, **b**) Time-course changes in total activity during awakening (**a**) and (**b**) sleeping periods. Changes in the ratio of the total activity to the mean value during the week before MIA administration (baseline) were calculated and plotted over time in each group. Asterisks indicate that the differences were statistically significant (*p* < 0.05) by *t*-test. (**c**, **d**) Time course changes of body temperature using nanotag. (**c**) The changes of body temperature during the awakening period were plotted over time. Pre inj indicates the mean value during the week before MIA administration. (**d**) The changes of body temperature during the sleeping period were plotted over time. Pre inj indicates the mean value during the week before MIA administration. Asterisk indicate *P* < 0.05. Student *T*-test was utilized for the statistical evaluation.

Figure 2a,b showed the changes in the amount of locomotor activity associated with knee inflammation in rats and accurately monitor the recovery of locomotor activity after intervention with BM. As shown in Fig. 2a, the total amount of locomotor activity during the awakening period (from 8PM to 8AM) decreased by almost 50% after the intra-articular injection of MIA, which was obviously larger than that of non-inflammatory control (PBS-PBS, Supplementary Fig. 1b). The total amount of locomotor activity remained low throughout the experimental period in the MIA-PBS group (Fig. 2a Red line, numerical data were shown in Supplementary Table 1). In the MIA-BM group, locomotor activity significantly increased after BM injection on day 4 and continued to increase throughout the experimental period compared with the MIA-PBS group (Fig. 2a Blue line). During the sleeping period (8AM to 8PM), we observed similar results. However, the analgesic effects of BM were relatively small (< onefold) during the experimental period (Fig. 2b).

Because the Nanotag can automatically record body temperature every 5 min, we next analyzed whether changes in body temperature could be used to assess the severity of knee pain in rats. Figure 2c,d and Supplementary Fig. 1d,e showed the time-course changes in body temperature in the three groups. As indicated, the body temperature during both the awakening and sleeping periods did not change significantly during the experimental period.

### Validation of pain measurement with Nanotag

In order to validate the use of Nanotag-based locomotor activity measurements for pain assessment, we compared the Nanotag data with histological findings and the results of Incapacitance tests.

**Figure 3:**
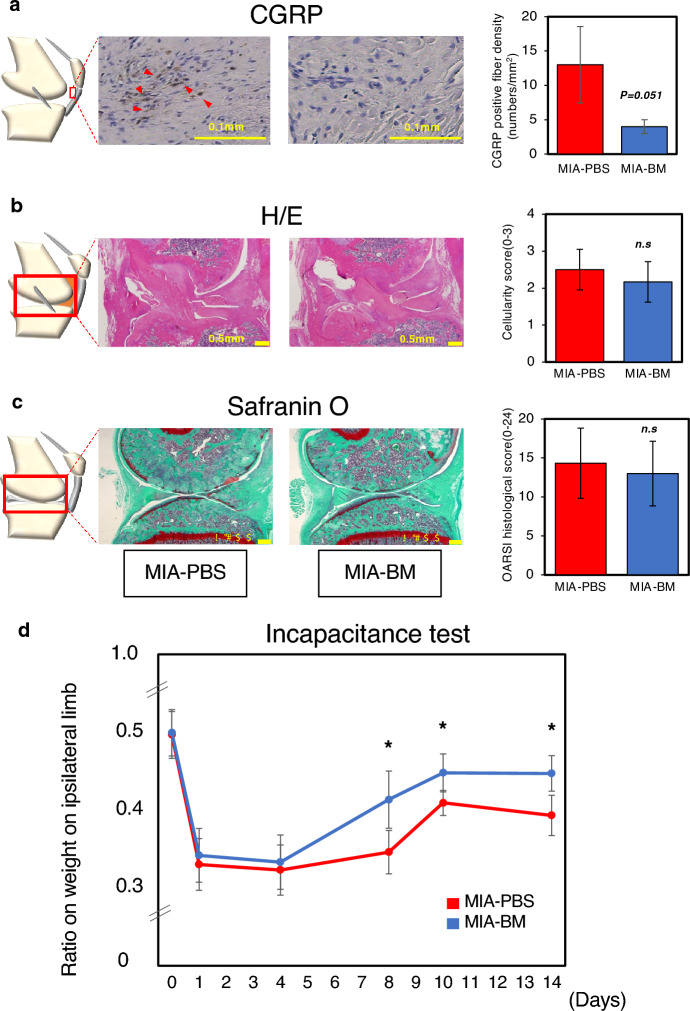
Histological and immunohistochemical evaluations of the knee joint of MIA-PBS and MIA-BM groups, and verification of reproducibility of measurement results with incapacitance test and Nanotag. (**a**) Immunohistochemical examination of IFP. (Left panel) A representative image of CGRP. Red arrowheads indicate CGRP-positive nerve fibers. (Right panel) The Number of CGRP-positive nerve fibers was counted in the IFP region and plotted (n = 3). Evaluations were performed by three independent researchers in a blinded manner. Inter-experimenter co-efficiency (ICC2.1) was 0.95 (95% CI 0.82–0.99)^15,16^. Data are shown as means ± SD. Student *T*-test was utilized for the statistical evaluation. (**b**) (Left panel) Mid sagittal sections of the knee joint were prepared and stained with hematoxylin and eosin (HE). (Right panel) In order to semi-quantitatively assess the severity of synovial inflammation, the IFP inflammation grading was determined according to the method described by Udo^17^ (n = 6). Evaluations were performed by three independent researchers in a blinded manner. Inter-experimenter co-efficiency (ICC2.1) was 0.73 (95% CI 0.45–0.90)^15,16^. Data are shown as means ± SD. Student *T*-test was utilized for the statistical evaluation. (**c**) (Left panel) Sagittal sections of medial condyle at intervals of 200 µm were stained with Safranin-O/Fast green and subjected for evaluation. (Right panel) Cartilage degeneration was evaluated according to the OA Research Society International (OARSI) grading system, as previously reported (n = 6)^17,18^. Evaluations were performed by three independent researchers in a blinded manner. Inter-experimenter co-efficiency (ICC2.1) was 0.95 (95% CI 0.87–0.98)^15,16^ Data are shown as means ± SD. Student *T*-test was utilized for the statistical evaluation. (**d**) Incapacitance test: The weight distribution of the ipsilateral hind limb was evaluated over time in both the MIA-PBS and MIA-BM groups (n = 12 in each group). Asterisks indicate that the differences were statistically significant (*p* < 0.05) by *t*-test.

Histological evaluation of the experimental subjects on day 15 revealed that the number of calcitonin gene-related peptide (CGRP)–positive fibers, which indicate sensitized nerve ends, was statistically non-significant (*P* = 0.051), yet noticeably lower in the MIA-BM group (Fig. 3a), whereas subtle differences in synovial inflammation (Fig. 3b) and cartilage degeneration (Fig. 3c) were observed between the groups on day 15.

We next compared the Nanotag results with data obtained using a weight-bearing test that is widely used to quantify pain and found that the changes in the weight-bearing ratio over time showed a trend similar to that of the data collected using Nanotag (Fig. 3d, compare it with Fig. 2a).

### Isolation-induced loss of locomotor activity in rats

**Figure 4:**
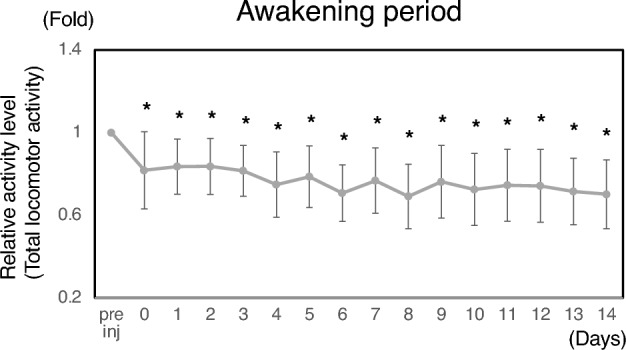
Isolation-induced loss of locomotor activity in rats. Time course changes of total locomotor activity during the awakening periods of isolated rats. The experimental procedure was performed in accordance with Supplementary Fig. 1a (PBS-PBS). In this experiment, each rat was housed separately. The changes in the ratio of total locomotor activity to the mean value during the week before PBS administration at day 0 were calculated and plotted over time (n = 20). Numerical data are presented in Supplementary Table 1. Asterisks indicate *P* < 0.05. Paired *T*-test was utilized for the statistical evaluation (each time point vs pre inj).

Emotional effects are considered to affect the amount of daily locomotor activity especially in social animals. Since Nanotag can measure locomotor activity in non-interventional way, we considered that this method would facilitate the quantification of the effects of social isolation-induced depression on daily locomotor activities. To test this idea, each rat was housed separately and had intra-articular injection of PBS on days 0, 4, 6, and 8 (PBS-PBS). Time course changes of total locomotor activity during the awakening periods of isolated rats were indicated in Fig. 4. As shown in this figure, the changes in the ratio of total locomotor activity to the mean value during the week before PBS administration at day 0 were gradually decreased with time.

### Joint inflammation induces a decrease in exercise intensity per unit time

**Figure 5:**
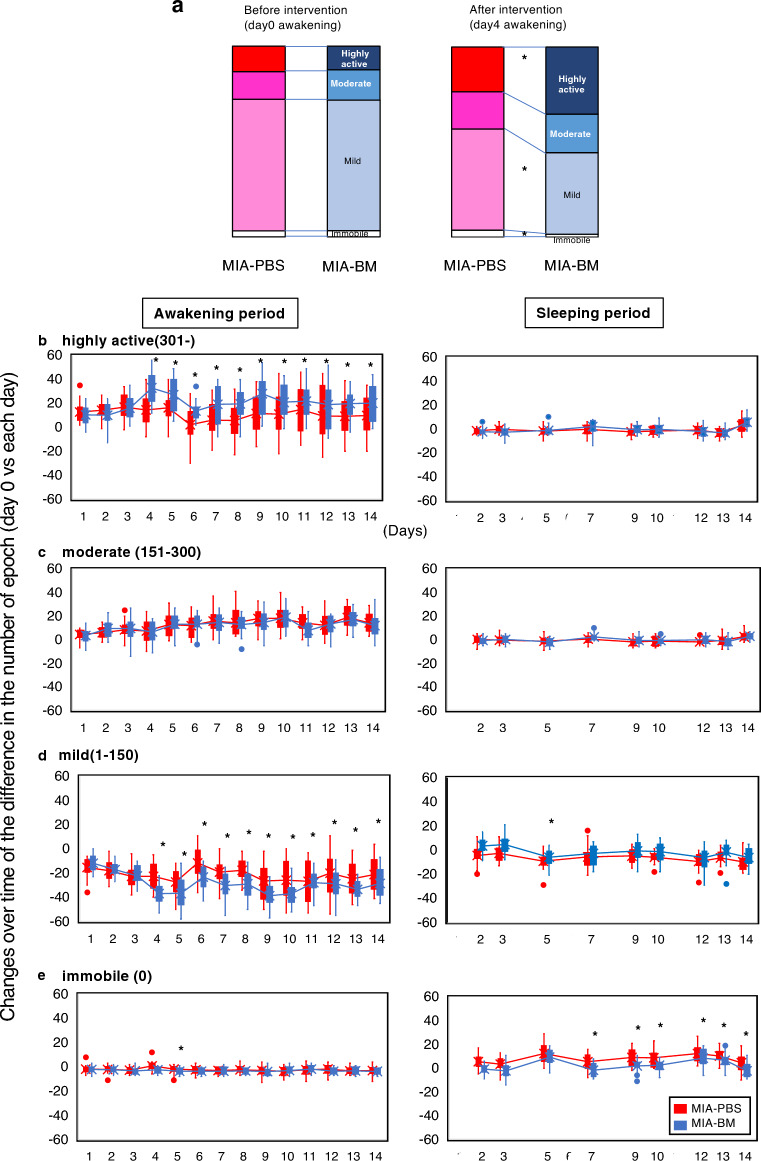
Measurement of locomotor intensity using Nanotag. (**a**) The ratios of highly active, moderate, mild, and immobile intensities before (day 0) and after (day 4) the intervention were calculated and plotted. Asterisks indicate *P* < 0.05, as determined by the *t*-test. (**b**–**e**) Changes over time of the difference in the number of epochs of highly active, moderate, mild, and immobile intensity ((awakening period):day 0 vs. each day, (sleeping period):day 1 vs. each day). Asterisks indicate *P* < 0.05, as determined by the *t*-test.

We also used the Nanotag device to analyze the patterns (intensity) of locomotor activity. We defined locomotor activity thresholds of 300 and 150 every 5 min as per prior studies and classified the intensity of activity for each epoch as follows: immobile (0), mild (1–150), moderate (151–300), and highly active (> 300)^13^ (Fig. 5). We observed that, during the awakening period, the number of epochs of highly active intensity significantly increased after BM injection, whereas that of mild intensity reciprocally decreased (Fig. 5a,b,d). The number of immobile and moderate-intensity epochs did not change significantly, regardless of the analgesic intervention (Fig. 5c,e). During the sleeping period, the locomotor activity at each intensity was low and showed subtle changes throughout the experimental period (Fig. 5).

### Pain measurement methods involving physical intervention have a significant impact on the daily locomotor activity in rats

**Figure 6:**
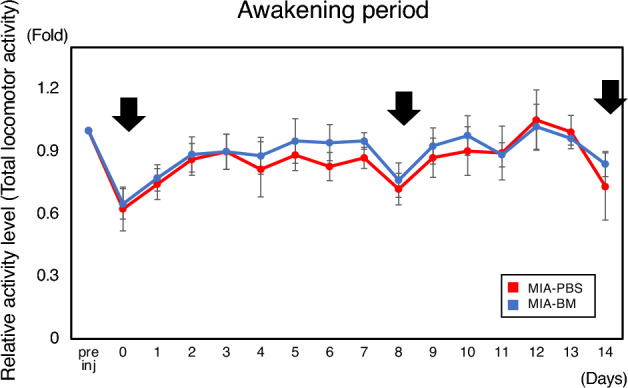
Pain measurement method involving physical intervention has a significant impact on the daily locomotor activity in rats. The changes in the ratio of total activities to the mean value during the week before MIA administration (baseline) were calculated using nanotag and plotted over time in each group (Blue: MIA-BM group (n = 6), Red: MIA-PBS group, n = 6). Incapacitance test was performed on days 0, 8 and 14. As indicated, we observed significant impact on total locomotor activity when the physical intervention measurement was performed (arrows). Note that this figure shows the amount of locomotor activity during the awakening period, however, the measurement with physical intervention was performed half a day earlier (Sleeping period).

To test if interventional behavioral tests affect locomotor activity (Nanotag results), incapacitance test was performed on days 0, 8 and 14 in rats with Nanotag monitoring. As indicated in Fig. 6, we observed significant impact on total locomotor activity when these physical intervention measurements were performed (arrows). Note that this figure shows the amount of locomotor activity during the awakening period, however, the measurements with physical intervention were performed half a day earlier (Sleeping period).

## Discussion

Experimental animal models are essential for analyzing molecular mechanisms of pain development and persistence, and various pain assessment techniques have been developed and studied in these models^1,2^. However, the quantitative evaluation of pain in experimental animals remains challenging. We posited that a knee pain assessment method meeting the following criteria would be beneficial: (1) complete objectivity, (2) availability for a wide range of species, (3) enabling continuous measurement throughout the day and night (in general, rodents are awake at night), (4) enabling simple data interpretation, (5) time effectiveness, (6) cost effectiveness, (7) clinical applicability in the future, (8) minimal stress for animals during measurement, (9) easy measurability without the need for adaptation or training for animals, and (10) applicability to group housing. Based on these considerations, the advantages and disadvantages of knee pain evaluation techniques in animals currently used by researchers are summarized in Table 1. From these comparative analyses of diverse measurement methods currently utilized in research laboratories, we have arrived at the conclusion that the implementation of Nanotag-based assessment confers notable advantages over these methods (Table 1).

**Table 1:** Experimental techniques to evaluate knee joint pain in rodents.

Inspection method	Methodological characteristics								Intervention to animal		
									Physical intervention	Emotional intervention	
	Description	Objective or subjective	Availability for wide range of species	Continuous or intermittent	Data interpretation	Time effectiveness (1)	Cost effectiveness (2)	Clinically applicable to evaluate knee pain?	Stress on animals during measurement	Need adoptation or training before starting experiment?	Social Isolation effects (Applicability to group housing)
*(1) Static measurement*											
(1–1) Stimulus induced pain											
Von frey hair test (Tactile hypersensitivity)	In ascending order of force, calibrated nylon filaments of different thicknesses are applied to the paw's plantar surface skin	Subjective	**Available**	Intermittent	**Simple**	Not effective	**Effective**	Not applicable	Stressful	Required	**Applicable**
Vocalizations elicited by knee extention test	Apply a knee extension to both the healthy and affected knees, or passive extention of the operated knee joint while sedated	Subjective	**Available**	Intermittent	**Simple**	Not effective	**Effective**	Not applicable	Stressful	Required	**Applicable**
Hotplate test (Thermal sensitivity)	Placed on a hotplate. Cutoff latencies are determined to avoid lesions	Subjective	**Available**	Intermittent	**Simple**	Not effective	**Effective**	Not applicable	Stressful	Required	**Applicable**
(1–2) neuromuscular screening											
Righting ability	Placed on its back	Subjective	**Available**	Intermittent	**Simple**	**Effective**	**Effective**	**Applicable**	Stressful	Required	**Applicable**
Cotton swab	Swab contact with eyelashes and whiskers, and pinna	Subjective	**Available**	Intermittent	**Simple**	**Effective**	**Effective**	Not applicable	Stressful	Required	**Applicable**
*(2) Spontaneous pain behavior (Nonstimulus)*											
Home cage monitoring (including video recordings)	The cages must be arranged in a specified platform one by one	**Objective**	Not available	**Continuous**	Complex	Not effective	Not effective	**Applicable**	**Not stressfull**	Required	Not applicable
Open field anaylysis	Experiments are conducted in transparent chambers and freely explored	**Objective**	Not available	Intermittent	Complex	Not effective	Not effective	**Applicable**	**Not stressfull**	Required	Not applicable
Gait analysis (Cat walk, foot print)	Automated quantitative gait analysis in dedicated cages equipped with fluorescent tubes and glass plates	**Objective Objective**	**Available**	Intermittent	Complex	Not effective	Not effective	**Applicable**	**Not stressfull**	Required	**Applicable**
Dynamic weight bearing (Incapacitance test)	Placed in a specific cage. This method involves measuring capacitance using a computer (similar to gait analysis)	**Objective**	Not available	Intermittent	**Simple**	Not effective	Not effective	**Applicable**	**Not stressfull**	Required	**Applicable**
Spontaneous wheel running	Placed in a specific cage with free access to stainless steel wheels. The wheel is linked to a computer and data is automatically recorded	**Objective**	Not available	**Continuous**	**Simple**	Not effective	**Effective**	Not applicable	**Not stressfull**	Required	Not applicable
Burrowing analysis	Placed in a specific cage made of plexiglas with steel tubes and quartz sand. Amount of sand burrowed is evaluated	Subjective	Not available	Intermittent	Complex	Not effective	**Effective**	Not applicable	**Not stressfull**	Required	Not applicable
*Challenged activity*											
Rotarod test	A rotating rod with a mouse on it is gradually accelerated	**Objective**	Not available	Intermittent	**Simple**	Not effective	**Effective**	Not applicable	Stressful	Required	**Applicable**
Hind limb and fore grip strength	Placed in front of a connected grasping device, over a base plate	**Objective**	Not available	Intermittent	**Simple**	Not effective	**Effective**	Not applicable	Stressful	Required	**Applicable**
Wire hang analysis	Suspended on a wire and time is measured	**Objective**	Not available	Intermittent	**Simple**	Not effective	**Effective**	Not applicable	Stressful	Required	**Applicable**
Nanotag	An implantable actimeter to measure both the locomotor activity of laboratory animals and their body temperature	**Objective**	**Available**	**Continuous**	**Simple**	**Effective**	Not effective	**Applicable**	**Not stressfull**	Required	**Applicable**

This table describes the pros and cons of pain evaluation techniques in animals currently utilized by researchers. In this table, items considered to be of “pros” are highlighted in Bold. As indicated in the table, all of the experimental techniques but not Nanotag have competing pros and cons, thus we consider that standardized pain analysis methods have yet to be established. (1) Time effectiveness: More than 20 animals are able to subject the analyses in a same day. (2) Cost effectiveness: The cost of conducting experiments generally below $100 per animal for acquiring data in an actual experiment.

In this study, we showed that Nanotag could detect the decrease in locomotor activity quickly after the arthritis induction and anti-inflammation analgesic treatment significantly increased it. These changes were in the same line with those of a conventional knee pain evaluation method, such as incapacitance test. These data indicated that Nanotag can be utilized as the non-interventional, continuous, and completely objective monitoring the amount of pain in rat knee arthritis model as the replacement of those conventional knee pain evaluation methods.

This study introduces a novel analytical approach for pain assessment, which involves analyzing locomotor activity according to the levels of exercise intensity. We defined locomotor activity as immobile, mild, moderate, and highly active, based on the activity counts (epochs) in every 5 min. Here we showed that, during the awakening period, the number of epochs of highly active intensity significantly increased after BM injection, whereas that of mild intensity reciprocally decreased. However, the number of immobile and moderate-intensity epochs did not change significantly, regardless of the analgesic intervention. These data indicate that pain alleviation using analgesics is specifically effective in increasing highly active locomotor activity during the awakening period.

Incapacitance test and von Frey Hair test are the measurement methods commonly used for pain assessment so far, however, these intervention-based measurement manipulations themselves have not been considered with respect to their emotional and physical effects on the amount of pain perception in animals. In this study, we demonstrated that Incapacitance test have a prolonged impact on subsequent locomotor activity. This data further suggest the superiority of non-interventional methods for pain evaluation.

It is considered that changes in body temperature can be one of the parameters for the perception of pain in animals. Since Nanotag can continuously monitor the body temperature, we also analyzed time course changes of body temperature in this study. However, as indicated in Fig. 2c,d, and supplementary Fig. 1d,e, changes in body temperature were subtle during the experimental period and was not in the same line with the time course changes in locomotor activity. These results could be improved by upgrading the performance of the temperature sensor or by reconsidering the location of implantation of Nanotag. From these data, we consider that significant procedural improvements are prerequisite to use the results of monitoring temperature changes for pain assessment in animals.

The strength of knee pain assessment using Nanotag is its complete non-invasiveness and objectivity, so that we can perform continuous 24 h measurements. In addition, this method enables us to analyze multiple experimental animals housed in the same cage without the necessity of installing specialized observation equipment to start up the experiment.

Limitation of this study is as follows. Firstly, Nanotag can measure locomotor activity in all directions (X-Y-Z) of the animal. However, it cannot provide detailed data used for evaluating hind limb pain, such as deviations in body axis during walking, changes in balance during stance and swing phases, pressure changes in the paw, alterations in knee flexion angle, and other specific parameters obtained through gait analysis. Therefore, it is still insufficient as an analytical method specialized in knee pain. Secondly, this device is currently unsuitable for long term study (unable to measure for more than 60 days) due to the limit of power supply. Thirdly, we used only male rats in this study. Since sex differences in pain sensitivity are widely recognized, we believe it is necessary to conduct similar experiments using female rats.

In summary, we showed that Nanotag can be utilized as the non-interventional, continuous, and completely objective monitoring the amount of pain in rat knee arthritis model. This traditional yet innovative method may be universally applicable to various pain models and species, making it a worthwhile device for research across diverse fields.

### Supplementary Information

Supplementary Figures.

Supplementary Tables.

## Data Availability

The datasets used and analyzed during the current study are available from the corresponding author on reasonable request.

## References

[1] Piel, M. J., Kroin, J. S., van Wijnen, A. J., Kc, R. & Im, H.-J. Pain assessment in animal models of osteoarthritis. *Gene* **537**, 184 (2014). 24333346 10.1016/j.gene.2013.11.091 PMC3950312

[2] Barrot, M. Tests and models of nociception and pain in rodents. *Neuroscience* **211**, 39–50 (2012). 22244975 10.1016/j.neuroscience.2011.12.041

[3] Bains, R. S. *et al.* Analysis of individual mouse activity in group housed animals of different inbred strains using a novel automated home cage analysis system. *Front. Behav. Neurosci.* **10**, 106 (2016). 27375446 10.3389/fnbeh.2016.00106 PMC4901040

[4] Klein, C. J. M. I. *et al.* Measuring locomotor activity and behavioral aspects of rodents living in the home-cage. *Front. Behav. Neurosci.* **16**, 877323 (2022). 35464142 10.3389/fnbeh.2022.877323 PMC9021872

[5] Mitchell, E. J., Brett, R. R., Armstrong, J. D., Sillito, R. R. & Pratt, J. A. Temporal dissociation of phencyclidine: Induced locomotor and social alterations in rats using an automated homecage monitoring system–implications for the 3Rs and preclinical drug discovery. *J. Psychopharmacol.* **34**, 709–715 (2020). 32438848 10.1177/0269881120920455 PMC7675779

[6] Russell, L. N. *et al.* Effects of laboratory housing conditions on core temperature and locomotor activity in mice. *J. Am. Assoc. Lab. Anim. Sci.* **60**, 272–280 (2021). 33888181 10.30802/AALAS-JAALAS-20-000093 PMC8145121

[7] Hoshino, T. *et al.* Persistent synovial inflammation plays important roles in persistent pain development in the rat knee before cartilage degradation reaches the subchondral bone. *BMC Musculoskelet. Disord.* **19**, 291 (2018). 30115046 10.1186/s12891-018-2221-5 PMC6097215

[8] Inomata, K. *et al.* Time course analyses of structural changes in the infrapatellar fat pad and synovial membrane during inflammation-induced persistent pain development in rat knee joint. *BMC Musculoskelet. Disord.* **20**, 8 (2019). 30611247 10.1186/s12891-018-2391-1 PMC6320593

[9] Onuma, H. *et al.* Fibrotic changes in the infrapatellar fat pad induce new vessel formation and sensory nerve fiber endings that associate prolonged pain. *J. Orthop. Res.*10.1002/jor.24580 (2020). 31903621 10.1002/jor.24580

[10] An, J. S. *et al.* Inhibition of fibrotic changes in infrapatellar fat pad alleviates persistent pain and articular cartilage degeneration in monoiodoacetic acid-induced rat arthritis model. *Osteoarthr. Cartil.* **29**, 380–388 (2021). 10.1016/j.joca.2020.12.014 33388431

[11] Yilmaz, E. The evaluation of the effectiveness of intra-articular steroid, tenoxicam, and combined steroid–tenoxicam injections in the treatment of patients with knee osteoarthritis. *Clin. Rheumatol.* **38**, 3243–3252 (2019). 31243588 10.1007/s10067-019-04641-y

[12] Yavuz, U., Sökücü, S., Albayrak, A. & Öztürk, K. Efficacy comparisons of the intraarticular steroidal agents in the patients with knee osteoarthritis. *Rheumatol. Int.* **32**, 3391–3396 (2012). 22057944 10.1007/s00296-011-2188-0

[13] Funabashi, D., Wakiyama, Y., Muto, N., Kita, I. & Nishijima, T. Social isolation is a direct determinant of decreased home-cage activity in mice: A within-subjects study using a body-implantable actimeter. *Exp. Physiol.* **107**, 133–146 (2022). 34921441 10.1113/EP090132

[14] Yu, D. *et al.* The inhibition of subchondral bone lesions significantly reversed the weight-bearing deficit and the overexpression of CGRP in DRG neurons, GFAP and Iba-1 in the spinal dorsal horn in the monosodium iodoacetate induced model of osteoarthritis pain. *PLoS ONE* **8**, e77824 (2013). 24204985 10.1371/journal.pone.0077824 PMC3813732

[15] Shrout, P. E. & Fleiss, J. L. Intraclass correlations: Uses in assessing rater reliability. *Psychol. Bull.* **86**, 420–428 (1979). 18839484 10.1037/0033-2909.86.2.420

[16] Koo, T. K. & Li, M. Y. A guideline of selecting and reporting intraclass correlation coefficients for reliability research. *J. Chiropr. Med.* **15**, 155 (2016). 27330520 10.1016/j.jcm.2016.02.012 PMC4913118

[17] Udo, M. *et al.* Monoiodoacetic acid induces arthritis and synovitis in rats in a dose-and time-dependent manner: Proposed model-specific scoring systems. *Osteoarthr. Cartil.* **24**, 1284–1291 (2016). 10.1016/j.joca.2016.02.005 26915639

[18] Pritzker, K. P. H. *et al.* Osteoarthritis cartilage histopathology: grading and staging. *Osteoarthr. Carti.* **14**, 13–29 (2006). 10.1016/j.joca.2005.07.014 16242352

[19] Kanda, Y. Investigation of the freely available easy-to-use software ‘EZR’ for medical statistics. *Bone Marrow Transpl.* **48**, 452–458 (2013). 10.1038/bmt.2012.244 PMC3590441 23208313

