# Supplementary material for: A novel methodology utilizing microchip implants to monitor individual activity and body temperature for assessing knee pain in group-housed rats
Source: Sci Rep. 2024 Jul 23;14:16909. doi: 10.1038/s41598-024-67024-7 (PMC11266718; doi:10.1038/s41598-024-67024-7)
Supplement: Supplementary file 1 — Supplementary Figures. [file 41598_2024_67024_MOESM1_ESM.pdf]

Supplementary Figures

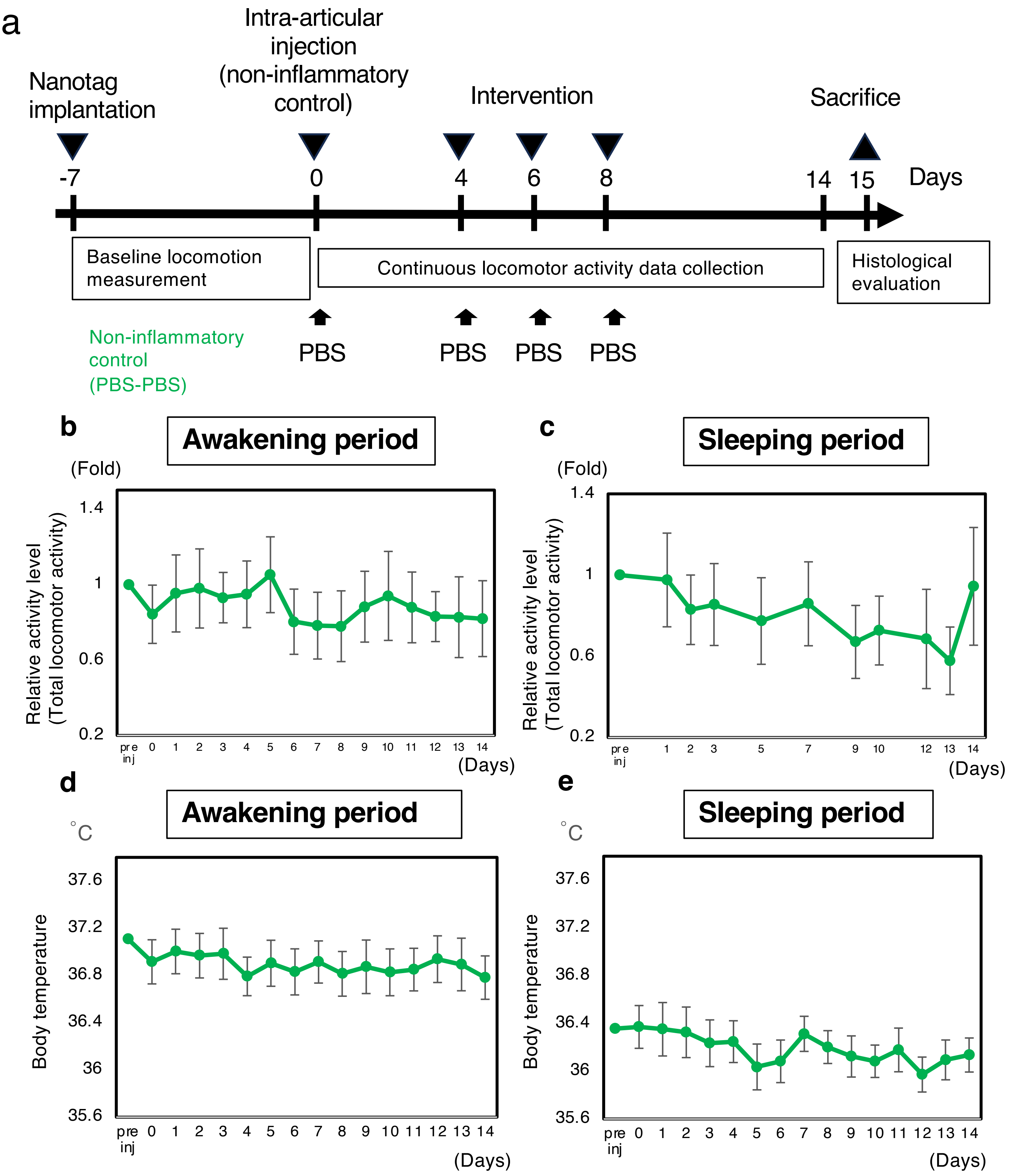

Supplementary Fig. 1 Experimental design and the results of behavioral and histological evaluations of non-inflammatory control group (PBS-PBS)

(**a**) Schematic diagrams of the experimental design of non-inflammatory control group (PBS-PBS). Nanotag implantation was performed 1 week before intra-articular injection of PBS as a negative control of joint inflammation. As a negative control of analgesic intervention, PBS-PBS group had PBS injection at days 4, 6, and 8 (n=18).

(**b**) Time course changes of total locomotor activity during the awakening periods of PBS-PBS group. The changes in the ratio of total locomotor activity to the mean value during the week before PBS administration at day 0 (pre inj) were calculated and plotted over time (n=18). Numerical data are presented in **Supplementary Table 1**.
(**c**) Time course changes of total locomotor activity during the sleeping periods. The changes in the ratio of total locomotor activity to the mean value during the week before PBS administration at day 0 (pre inj) were calculated and plotted over time (n=18). Numerical data are presented in **Supplementary Table 1**.
(**d**) Histological and Immunohistochemical evaluations of the knee joint at day 15. Representative pictures are indicated.
(**Left panel**) Mid sagittal sections of the knee joint were prepared and stained with hematoxylin and eosin. In order to semi-quantitatively assess the severity of synovial inflammation, the IFP inflammation grading was determined according to the method described by Udo et al.[1] (n=6). Evaluations were performed by three independent researchers in a blinded manner. Inter-experimenter co-efficiency (ICC2.1) was 0.81 (95% CI 0.45-0.97)[2,3].
(**Middle panel**) Cartilage degeneration was evaluated according to the OA Research Society International (OARSI) grading system, as previously reported[1,4]. Sagittal sections of medial condyle at intervals of 200 μm were stained with Safranin-O/Fast green and subjected for evaluation (n=6). Evaluations were performed by three independent researchers in a blinded manner. Inter-experimenter co-efficiency (ICC2.1) was 0.91 (95% CI 0.71-0.99)[2,3].
(**Right panel**) The mid-sagittal sections were subjected to immunohistochemical staining for CGRP-positive nerve fibers. The number of CGRP-positive fibers was counted according to established methods[5–7]. Immunohistochemical assessments were performed by three independent researchers in a blinded manner. Inter-experimenter co-efficiency (ICC2.1) was 0.95 (95% CI 0.72-1.00)[2,3].

1. Udo, M. et al. Osteoarthritis and Cartilage 24, 1284–1291 (2016).
2. Shrout, P. E. & Fleiss, J. L. Psychological bulletin 86, 420–428 (1979).
3. Koo, T. K. & Li, M. Y. Journal of Chiropractic Medicine 15, 155 (2016).
4. Pritzker, K. P. H. et al. Osteoarthritis and Cartilage 14, 13–29 (2006).
5. Hoshino, T. et al. BMC Musculoskelet Disord 19, 291 (2018).
6. Onuma, H. et al. Journal of Orthopaedic Research jor.24580 (2020)
7. An, J. S. et al. Osteoarthritis and Cartilage 29, 380–388 (2021).

a

Day		-7	-6	·····	-1	0	1	2	·····	14
Time		Activity counts in every 5 min								
PM	8:00	47	77	·····	407	93	71	2	·····	99
	8:05	162	55	·····	977	385	380	12	·····	279
	8:10	25	366	·····	449	597	664	3	·····	462
	⋮	⋮	⋮	·····	⋮	⋮	⋮	⋮	·····	⋮
	11:50	8	59	·····	680	35	1	559	·····	432
	11:55	118	72	·····	605	195	54	74	·····	562
AM	0:00	39	63	·····	407	7	78	80	·····	487
	0:05	104	49	·····	140	16	75	694	·····	264
	0:10	203	90	·····	59	132	289	316	·····	319
	⋮	⋮	⋮	·····	⋮	⋮	⋮	⋮	·····	⋮
	7:55	8	60	·····	60	5	124	77	·····	81
Total activity counts		35521	44138	·····	40213	17240	22488	27213	·····	35994
						B_0	B_1	B_2	·····	B_{14}

Average of total activity counts (Awakening period) Ⓐ

Relative activity level (each day) = B_n / Ⓐ

Ear Tag No. 418

Ear Tag No. 419

Ear Tag No. 420

Ear Tag No. 421

....

b

Day		0		1		2		·····	14	
Time		Counts	Intensity	Counts	Intensity	Counts	Intensity	·····	Counts	Intensity
PM	8:00	93	Mild	71	Mild	2	Mild	·····	99	Mild
	8:05	385	Highly active	380	Highly active	12	Mild	·····	279	Moderate
	8:10	597	Highly active	664	Highly active	3	Mild	·····	462	Highly active
	⋮	⋮	⋮	⋮	⋮	⋮	⋮	·····	⋮	⋮
	11:50	35	Mild	1	Mild	559	Highly active	·····	432	Highly active
	11:55	195	Moderate	54	Mild	74	Mild	·····	562	Highly active
AM	0:00	7	Mild	78	Mild	80	Mild	·····	487	Highly active
	0:05	16	Mild	75	Mild	694	Highly active	·····	264	Moderate
	0:10	132	Mild	289	Moderate	316	Highly active	·····	319	Highly active
	⋮	⋮	⋮	⋮	⋮	⋮	⋮	·····	⋮	⋮
	7:55	5	Mild	124	Mild	77	Mild	·····	81	Mild

Number of occurrences of each activity intensity (Number of epochs during awakening period)

Day	0	1	2	·····	14	Difference in the number of epochs
Immobile (0)	A_0 5	A_1 1	A_2 0	·····	A_{14} 1	$A_n - A_0$
Mild (1 - 150)	B_0 99	B_1 89	B_2 87	·····	B_{14} 54	$B_n - B_0$
Moderate (151 - 300)	C_0 23	C_1 31	C_2 17	·····	C_{14} 37	$C_n - C_0$
Highly active (> 301)	D_0 17	D_1 23	D_2 40	·····	D_{14} 52	$D_n - D_0$

Ear Tag No. 418

Ear Tag No. 419

Ear Tag No. 420

Ear tag No. 421

...

c

Day		-7	-6	·····	-1	0	1	2	·····	14
Time		Body temperature in every 5 min								
PM	8:00	36.5	36.2	·····	36.4	36.1	36.3	36.5	·····	35.8
	8:05	36.7	36.1	·····	36.9	37.0	36.3	36.9	·····	35.7
	8:10	36.6	35.9	·····	36.9	37.4	36.9	36.6	·····	36.4
	⋮	⋮	⋮	·····	⋮	⋮	⋮	⋮	·····	⋮
	11:50	36.9	37.0	·····	37.3	36.3	35.8	36.8	·····	36.9
	11:55	36.9	36.9	·····	37.3	36.6	36.3	36.6	·····	37.3
AM	0:00	36.9	36.8	·····	37.3	36.8	36.4	36.5	·····	37.3
	0:05	37.1	36.7	·····	37.0	37.2	36.9	36.8	·····	36.5
	0:10	37.2	36.5	·····	36.7	36.8	36.9	36.9	·····	36.8
	⋮	⋮	⋮	·····	⋮	⋮	⋮	⋮	·····	⋮
	7:55	36.4	35.9	·····	36.3	37.1	36.6	36.6	·····	36.3
Average		37.0	36.8	·····	36.8	36.8	36.6	36.6	·····	36.5

Ⓐ pre injection (Average of body temperature from day-7 till day-1)

Ear Tag No. 418

Ear Tag No. 419

Ear Tag No. 420

Ear Tag No. 421

...

Hasegawa et al. Supplementary Fig2

Supplementary Fig. 2 Data analysis (Locomotor activity counts, intensity, and body temperature)

(**a**) Evaluation of total locomotor activity counts during the awakening and the sleeping period (**Fig. 2a, 2b, 4, 6 and Supplementary Fig. 1b, 1c**)
To evaluate relative activity levels between the experimental groups, the total activity counts in every 5 min in each rat was calculated as follows. Data collection was performed according to the schedule described in **Fig. 1c**. Data were analyzed separately for the awakening period (from 8PM till 8AM) and the sleeping period (from 8AM till 8PM) as described in **Fig. 1d**.
(1) Calculation of baseline value (pre inj)
Since large individual differences in basal activity counts between rats were observed, daily activities were normalized using the average of daily activity counts for the week from Nanotag implantation (day-7) to the day before joint inflammation induction (day-1) as indicated in **Fig. 1c**.
(2) Calculation of relative activity level
Relative activity level of each rat was calculated using the equation indicated below.
[Relative activity level (fold) = Total activity counts (Bn)/pre inj (A)]
Relative activity level was calculated separately in each rat. Data are presented as mean and SD values of all rats. Numerical data are presented in **Supplementary Table 1**.

(**b**) Classification of activity intensity and assessment of changes over time in each activity intensity (difference in the number of epochs from day 0 (Awakening period; day 0 vs each day) or from day 1 (Sleeping period; day 1 vs each day)) (**Fig.5 b-e**)
Since the nanotag device can record patterns of the locomotor activity, we defined locomotor activity thresholds of 300 and 150 every 5 minutes, as per prior studies and classified as follows: immobile (0), mild (1-150), moderate (151-300), and highly active (>300). The number of epochs of locomotor activity was then counted in each activity intensity. Intensity of activity for every 5 min was categorized as the definition and total epoch numbers of each category for 12 hrs were counted. Difference in the number of epochs from day 0 was calculated.

(**c**) Evaluation of time course changes in body temperature
Body temperature of each rat was automatically recorded by every 5 min by Nanotags. Average body temperature was calculated as mean and standard deviation, and plotted in **Fig. 2c, 2d, and Supplementary Fig. 1d, 1e**.
